# Designing Vermont’s Pay-for-Population Health System

**Published:** 2010-10-15

**Authors:** James Hester

**Affiliations:** Health Care Reform Commission, Vermont State Legislature

## Abstract

Vermont is developing a health care system that could offer a unique opportunity to test a new model for improving population health. Four lines of development converged for the system: 1) a published challenge to create a pay-for-population health system, 2) comprehensive state health reform legislation, 3) the Institute for Healthcare Improvement Triple Aim project, and 4) the concept of the accountable care organization (ACO). In phase 1 of pilot testing, 3 communities serving 10% of the population are using the system, which is based on the *enhanced medical home* model. Planning is under way for phase 2 of the pilot, ACOs that use incentives based on the Triple Aim goals. Vermont has created a conceptual framework for a community health system and identified some of the practical issues involved in implementing this framework.

This article summarizes the design and implementation of the enhanced medical home pilots and the results of a feasibility study for the ACO pilots. It describes how one state is using a systematic approach to health care reform to overcome some of the implementation barriers to a pay-for-population health system. Vermont will continue to provide a statewide laboratory for a pay-for-population health system.

## Introduction

Since 2006, 4 lines of development have converged in Vermont's health care reform program, creating a unique opportunity to test a new model for improving population health. First, more than a decade ago, Kindig (1) issued a challenge to improve the outcomes of an American health care system that spends twice as much per capita for health care services as other developed countries, while achieving third-world rates of illness and death. Recently, he renewed the challenge, calling for the development of a "pay-for-population health performance system that goes beyond medical care to include financial incentives for the equally essential nonmedical care determinants of population health" (2).

Second, in 2006 Vermont enacted legislation creating one of the nation's most ambitious health care reform programs (3). Building on foundations laid in the previous 5 years, the state attempted to achieve a sustainable reduction in the number of uninsured residents, accelerate the implementation of health information technology, and transform the prevention and treatment of chronic illness through a program called Blueprint for Health. Treatment of chronic illness accounts for more than 65% of all health care expenses in Vermont, but current practices offer major opportunities for improving performance. Blueprint for Health is based on the Chronic Care Model (4) and is a true public-private partnership supported by a broad base of stakeholders (5). Having only 600,000 residents, Vermont proved to be an ideal laboratory for testing meaningful delivery system reform as a major component of its broader health care reform effort to improve coverage and health information technology. Its small-scale, noncompeting delivery system and history of collaboration between stakeholders provided a supportive, nurturing environment for the proposed changes. Every year since the initial health reform legislation passed in 2006, Vermont has added legislation to strengthen and broaden health reform, including mandating a model called the *enhanced medical home* and coordinating strategies to prevent chronic illness. The legislature and its Health Care Reform Commission have led this process, but the implementation of delivery system reform has required sustained shared leadership by both the legislative and executive branches and by private-sector stakeholders.

Third, in 2007 the Institute for Healthcare Improvement began its Triple Aim project to drive large-scale system change by 1) controlling total per capita medical costs, 2) improving the population's health, and 3) improving the care experience of health care consumers (6). The institute created a learning collaborative that brought together an international collection of health care organizations implementing the Triple Aim project. The Vermont Blueprint for Health accepted the invitation to join the initial learning collaborative and continues to participate.

Finally, Vermont adopted the model of the accountable care organization (ACO) suggested by Fisher et al (7) based on their research documenting widespread, large variations in health care use without improvement in outcomes. The ACO model is built around creating a new set of financial incentives for a community provider network of physicians, local hospitals, and other caregivers for a defined population. The financial incentives are based on a pool of shared savings that is distributed when specific quality criteria are achieved.

This article describes Vermont's statewide effort, which weaves together these 4 lines of development and offers the prospect of creating a prototype for Kindig's pay-for-population health system in similarly rural areas. As part of its broader health care reform agenda, Vermont is attempting to build a statewide network of community health systems, which would provide both the infrastructure and financial incentives required to improve population health. The community health system involves multiple levels of reform to create the integration needed for effective population health incentives. The first, most basic, level is the enhanced medical home, which gives primary care practices the ability to better coordinate care with other providers and support behavior changes in their patients. The second level is the ACO, composed of the local hospital, specialists, and other key providers who work with the medical home practices. The Vermont community health model incorporates a prevention and population health incentive.

By the end of 2009, phase 1 of system reform was implemented in 3 pilot communities serving 10% of the state's population. Planning is under way for phase 2 pilot programs that combine the ACO concept with an incentive model built on the Triple Aim goals. Legislation enacted in May 2010 expands the enhanced medical home program from a pilot to a statewide initiative and commits state support to phase 2: 3 ACO pilots that use incentives based on the Triple Aim goals. Several characteristics make Vermont a unique statewide laboratory for implementing these reforms. It has a small population, a delivery system with no directly competing hospitals, a simple payer system with only 3 major commercial payers, and a long tradition of collaboration between major stakeholders. Health care reform has enjoyed long-term bipartisan support from both a Republican governor and a Democratic-majority state legislature. These qualities make it unlikely that other states will implement community health systems in exactly the same way that Vermont has, but the conceptual framework developed in Vermont can be generalized to other settings, particularly those with more rural delivery systems. This article will first present the conceptual framework of the proposed network of community health systems, focusing on the different types of integrator roles necessary for success. Then, it will describe the design of the enhanced medical home pilots and the results of the feasibility study for the ACO pilots.

## A Conceptual Framework for a Community Health System

The Vermont experience has revealed the necessity of integration at 3 geographic levels.

- **Enhanced medical home.** The National Committee for Quality Assurance (NCQA) defines the *patient-centered medical home* as a health care setting that facilitates partnerships between patients and their physicians through the use of registries, information technology, and health information exchange. This is the foundation level of integrating care to meet individual patient needs. The medical home is particularly challenging for small practices that must coordinate care across multiple settings and support patients through long-term behavioral changes. Because most Vermont primary care practices are small (fewer than 5 physicians), the Blueprint for Health uses an enhanced medical home model, which provides more support to small practices.
- **Community health system.** The ACO is 1 example of a community health system, what Fisher called the "neighborhood for the medical home" (8). The broader definitions of an ACO require only primary care physicians, but for Vermont, this geographic level must consist of at least a local health care provider network composed of a community hospital, its medical staff of primary care and specialist physicians, and other caregivers working within a geographic area that would typically be defined by the service area of the hospital. The community health system level needs to expand to include a broader array of public health and community resources for maintaining the health of a population. Large urban areas could have overlapping community health systems in the same region, which complicates their development. Fortunately, Vermont's rural quality means none of its 13 hospital service areas overlap.
- **Region or state.** The medical home and community health system levels depend on the creation of supporting infrastructure at a larger regional level. Some examples are health information technology support, such as regional health information exchange (secure, appropriate exchange of digital health information among providers and with patients); payment reforms; and technical support services and training programs to develop process improvement capacity and disseminate best practices. In Vermont, this supporting infrastructure has been implemented at the state level, but larger states may need to use regional structures.

The 3 geographic levels are interdependent, interacting through the following 5 categories of functional capacity that create the required integration.

- Service integration is necessary across levels and settings of care. Examples include patient-centered integrated care models at the patient level and integrated health care, public health, and social services that support population health at the community level.
- Financial integration refers to unified payments and incentives across multiple payers at the state level and local management of integrated budgets at the community level. Vermont used legislative mandates to require Medicaid and major commercial payers to participate in a common set of payment reforms to support delivery system transformation. The state could not mandate Medicare participation, but it used state funds to pay for Medicare's share of payment reforms so it could test all payer models in its pilots.
- Governance provides leadership and establishes accountability at the community level under a state-regulated framework.
- Process improvement refers to changes in clinical and administrative processes to improve performance at both the patient-centered medical home and community health system levels. This capability lies at the heart of a high-performing health system and requires engagement at all 3 geographic levels.
- Information tools include both information technology and reports to support care and to assess performance. Successful implementation of effective information tools requires mutually supportive efforts at all 3 geographic levels.

Vermont's reform plan consists of 2 phases, the first at the medical home level and the second at the community health system level. These reforms include changes in financial incentives to transform the delivery system; they have been challenging to design in a multipayer environment. Although payment reform is necessary, it is not a sufficient requirement for building a community health system. Too often, policy makers have assumed that simply changing the financial system will drive other necessary changes. Vermont's experience shows that the substantial structural changes needed require building new capabilities in all 5 functional categories.

To concentrate resources and coordinate efforts, Vermont used pilot communities. This approach had several benefits. First, because the changes were pilots and not systemwide, they were less threatening and easier to adopt. For example, it would have been impossible to implement all payment reforms statewide. Second, the competition to become a pilot community galvanized local leadership and created a more receptive climate for change. Third, scarce state resources could be focused in a more concentrated way, which prevented their premature dilution. Finally, the pilot design incorporated formative evaluations, which allowed the state to learn while implementing and recognize that these efforts are a work in progress. The corollary to the use of pilots is that scaling them statewide will require federal support through national health reform. Vermont can begin the process of building a community health system but cannot finish the task with state resources alone.

## Phase 1: The Enhanced Medical Home

The enhanced medical home pilots involve primary care practices in 3 communities (9). These pilots are designed to strengthen the functional capacity of primary care practices to coordinate care across settings and to support behavior changes in their patients while providing the infrastructure to enable them to serve as a medical home. The objective of the pilots is to reduce the prevalence of chronic illness and its complications and to improve compliance with national prevention and treatment guidelines. The pilots have 5 components.

- **Financial reform.** All major payers — the 3 major commercial insurers, Medicaid, and Medicare — must reform their payment systems. (To begin the program in a timely way, the state is paying the full incremental costs for Medicare patients, with the objective of obtaining federal support in 2010.) The payment reform features 2 elements ― a monthly per capita payment directly to each practice and the funding of a local community health team as a shared resource for multiple practices. The per capita payment is based on a semiannual assessment of each practice by outside evaluators using the NCQA Patient Centered Medical Home assessment tool (10). Each payer makes a monthly payment to the practice based on the score and the payer's panel size. For a physician with a panel of 2,000 patients, the maximum payment would be approximately $60,000 per year in addition to the usual fee-for-service payments.
- **Community health teams.** These are multidisciplinary teams that provide support and expertise to enhanced medical home practices through direct services, care coordination, population management of the patient panel (based on segmentation according to need), and quality improvement activities. Because community health teams are designed to meet the needs of their specific communities, the exact mix of resources varies. They typically include nurse care coordinators, behavioral health professionals, community health workers, and a prevention specialist from the district office of the Vermont Department of Health (a total of 5 full-time equivalent staff for a patient population of 20,000). Involving the prevention specialist in the community health team ensures that prevention programs are developed collaboratively by public health and health care delivery specialists, while maximizing program impact.
- **Health information technology.** A medical home is unlikely to function effectively without robust health information technology tools to identify patients with chronic illnesses, track their needs, and coordinate their care. The Blueprint for Health defined a core set of guideline-based data elements that are common across all sites, and each site enters those data into a Web-based clinical tracking system called the DocSite Registry (DocSite, LLC, Raleigh, North Carolina) that is used by all practices in pilot communities. DocSite captures data on all patients who are active with the practice. It can produce both visit planners to structure the activities for each patient visit and population-based reports at all 3 geographic levels. Participating practices have updated their electronic medical records to provide the core data elements to DocSite through statewide health information exchange. Practices have found DocSite essential for producing the population-based reports necessary to track patients and coordinate care.
- **Community activation and prevention.** Three tasks of the community health team are to complete a community risk profile, prioritize prevention interventions, and implement a local prevention plan in coordination with the delivery system. In developing the community risk profile, the community health team's prevention specialist is supported by state data sources, including vital statistics, hospital discharge data, census data, Behavioral Risk Factor Surveillance System data, and surveys of tobacco use prevalence. The pilot communities are merging elements from these databases to create multidimensional data sets capable of providing rich profiles on the health of the population. For example, the St. Johnsbury community health team has been collaborating with staff from the Dartmouth Population Health Research Center and the Triple Aim project to develop its population health measures. The team has created a local version of the drivers-of-health model developed by the University of Wisconsin that includes nonmedical determinants of health (11).
- **Evaluation.** The pilot programs will be comprehensively evaluated after 20 months using data sets that include the NCQA Patient-Centered Medical Home scores, clinical process measures, health status measures, cost and utilization measures from a multipayer claims database, and population health indicators. The patients in the pilot practices will be compared with a matched sample of patients outside of the pilot practices. The data collection for the evaluation has been built into the transaction support for the day-to-day operation of the pilots and is designed to have minimal additional impact.

## Phase 2: The Accountable Care Organization

If the phase 1 pilots achieve results similar to those of other closed-system settings such as the Geisinger Health System (12), they will be able to meet the first of the Triple Aim goals, per capita cost savings, by reducing unnecessary hospital admissions and emergency department visits for treatment of chronic disease. To meet the other 2 Triple Aim goals of improving population health and experience of care, the local community health system must be able to share in those savings and reinvest them locally. The community activation and prevention plan created by the pilot community health teams will guide investments in each community, including priorities for key nonmedical determinants of health. However, in the absence of a second phase of reform, the financial benefits of the enhanced medical home simply flow downstream to the payers. The primary care practices have received an enhanced payment, but otherwise the community has no additional resources available to improve the health of its population.

Vermont's ACO model incorporates the Triple Aim incentives to address this issue. The model creates a shared savings incentive pool based on projected medical expenses, which is distributed on the basis of agreed-on quality measures and population health targets. As the next stage of health system reform to build a sustainable community health system, ACO pilots will be implemented. The Health Care Reform Commission has conducted a feasibility study for implementing a community-level incentive system based on ACOs (13). At the same time, the Dartmouth Institute for Health Policy and Clinical Practice and the Englelberg Center for Health Care Reform at Brookings jointly developed a national learning collaborative to implement several ACO pilots nationwide. Staff from both organizations participated in the Vermont feasibility study and contributed their research findings. After finding encouraging results from this study, legislation was passed directing the Health Care Reform Commission to collaborate with the executive branch of the state government and interested provider networks to develop a Vermont application for the ACO national learning collaborative (14).

The ACO feasibility study created a working design for the pilots, building on the medical home as the essential first step (15). The Health Care Reform Commission created a broad-based work group that identified potential obstacles to building the community level of integration. The group focused on 3 categories.

- **The scope and scale of the pilot.** The scope of covered benefits included in the shared savings budget should be broad, encompassing not only physician and hospital care but also prescription drugs and behavioral health services. To have statistically meaningful medical expense budgets and savings, the minimum population for an ACO is 15,000 commercial members, 10,000 Medicaid members, or 5,000 Medicare members.
- **Functional responsibilities of an ACO and criteria for a community provider network to qualify.** To succeed as a system integrator, an ACO must possess the 5 functional capacities (financial reform, community health teams, health information technology, community activation and prevention, and evaluation). The pilots need to start with a local provider organization such as a physician-hospital organization with experience and a proven track record in most of these skills.
- **Financial model and the design of Triple Aim incentive measures.** The work group concluded that reasonable starting points for meaningful measures of all 3 Triple Aim goals (controlling total per capita medical costs, improving population health, and improving the care experience) were available. They explored in detail key issues in designing the financial model and setting total per capita cost targets. These efforts yielded a set of population measures that could be implemented in approximately 2 years, with the understanding that the measures would likely change rapidly after implementation.

Qualified ACO pilot sites were identified, and the Vermont ACO pilots are being developed. The state regulatory agency for insurance is facilitating conversations with commercial insurers regarding a shared savings pool. Vermont's state Medicaid agency is also developing a plan to participate in the ACOs. The Blueprint for Health program is contributing to the design of the ACO model to ensure effective coordination between the medical home practices and ACOs.

## Conclusions

Vermont has not yet created a true pay-for-population health system, but the state has found no obstacles that cannot be overcome. A substantial missing piece, federal participation, is not assured, but  national health care reform legislation explicitly authorizes and funds Medicare participation in ACO pilots. Vermont provides a statewide laboratory for assembling a bench model that will allow the state to test design issues that still need to be explored. Building a replicable, functioning pay-for-population health system should be just a matter of time.
